# Safety in the operating room during orthopedic trauma surgery—incidence of adverse events related to technical equipment and logistics

**DOI:** 10.1007/s00402-017-2862-0

**Published:** 2017-12-21

**Authors:** E. A. K. van Delft, T. Schepers, H. J. Bonjer, G. M. M. J. Kerkhoffs, J. C. Goslings, N. W. L. Schep

**Affiliations:** 1 Department of Surgery, VU Medical Center, 7057, Boelelaan 1117, 1007 MB Amsterdam, The Netherlands; 2 Department of Trauma surgery, AMC Amsterdam, Meibergdreef 9, 1105 AZ Amsterdam, The Netherlands; 3 Department of Orthopedic surgery, AMC Amsterdam, Meibergdreef 9, 1105 AZ Amsterdam, The Netherlands; 4 Department of Trauma surgery, Maasstad Hospital, Rotterdam, Maasstadweg 21, 3079 DZ Rotterdam, The Netherlands

**Keywords:** Surgical, Trauma, Orthopedic, Adverse events

## Abstract

**Background:**

Safety in the operating room is widely debated. Adverse events during surgery are potentially dangerous for the patient and staff. The incidence of adverse events during orthopedic trauma surgery is unknown. Therefore, we performed a study to quantify the incidence of these adverse events. Primary objective was to determine the incidence of adverse events related to technical equipment and logistics. The secondary objective was to evaluate the consequences of these adverse events.

**Methods:**

We completed a cross-sectional observational study to assess the incidence, consequences and preventability of adverse events related to technical equipment and logistics during orthopedic trauma surgery. During a 10 week period, all orthopedic trauma operations were evaluated by an observer. Six types of procedures were differentiated: osteosynthesis; arthroscopy; removal of hardware; joint replacement; bone grafting and other. Adverse events were divided in six categories: staff dependent factors; patient dependent factors; anaesthesia; imaging equipment; operation room equipment and instruments and implants. Adverse events were defined as any factor affecting the surgical procedure in a negative way.

**Results:**

Hundred-fifty operative procedures were included. In 54% of the procedures, at least one adverse event occurred. In total, 147 adverse events occurred, with a range of 1–5 per procedure. Most adverse events occurred during joint replacement procedures. Thirty-seven percent of the incidents concerned defect, incorrect connected or absent instruments. In 36% of the procedures adverse events resulted in a prolonged operation time with a median prolongation of 10.0 min.

**Conclusion:**

In more than half of orthopedic trauma surgical procedures adverse events related to technical equipment and logistics occurred, most of them could easily be prevented. These adverse events could endanger the safety of the patient and staff and should therefore be reduced.

**Level of evidence:**

4.

## Introduction

Adverse events frequently occur during medical treatment, between 44.000 and 98.000 patients are estimated to die each year in the U.S.A. as a result of medical errors [1]. At present, the exact incidence of adverse events in the operating room (OR) and their impact remains unclear. However, it is clear that many adverse events occur during surgery due to the complexity of an OR which depends on interrelation between staff, instruments and implants [2].

Over the last decennium the surgical environment has become more complex. New problems emerge in man-equipment interaction during high-tech procedures, thereby creating opportunities for errors or incidents to occur. The negative effects of distractions during surgery have been described by Weigl et al. He found that well designed operating room environment and awareness of the surroundings prevent interruptions and ensure effective and safe surgical care [3].

Since 2009 hospitals in the Netherlands are obliged to implement a checklist system to enhance surgical safety [4]. The SURPASS (SURgical PAtient Safety System) has been developed and implanted in our level 1 trauma center. The SURPASS is a multidisciplinary checklist covering the entire surgical pathway; such as preoperative checks, time-out procedure and postoperative reports. Implementation of the SURPASS resulted in a decrease of incidents (27.3–16.7%) and a decrease of in-hospital mortality (1.5–0.8%). The checklist includes information about the patient and the procedure (name, type of surgery, side of surgery) as well as the technical equipment (instruments and implants) [5]. Despite this checklist, adverse events during surgery still occur.

Adverse events during surgery are potentially dangerous for the patient and OR staff. The incidence of these adverse events, especially during orthopedic trauma surgical procedures, is unknown. Therefore, we performed a study to quantify the incidence of these adverse events. Primary objective was to determine the incidence of adverse events related to technical equipment and logistics. The secondary objective was to evaluate the consequences of these adverse events.

## Materials and methods

A cross-sectional observational study of all consecutive elective and acute orthopedic trauma operative procedures was performed during a 10 week period in September and October 2014 in an academic Level-1 trauma center, the Academic Medical Center Amsterdam. One observer in the operating room scored all adverse events and tracked their effect on the procedure and the patient. Surgical procedures conducted during night and evening (6PM–8AM) were excluded due to unavailability of an observer. Patients under 16 years were excluded as well.

Six types of procedures were differentiated: (1) Osteosynthesis; (2) Arthroscopy; (3) Removal of hardware; (4) Joint replacement; (5) Bone grafting and (6) other (e.g. repair of tendon ruptures, wound debridement). Adverse events were divided in six categories: (1) staff dependent factors; (2) patient dependent factors; (3) anaesthesia; (4) imaging equipment; (5) operation room equipment and (6) instruments and implants.

Data were analyzed with IBM SPSS Statistics for Windows, Version 22.0. Armonk, NY: IBM Corp. Released 2013.Normality of continuous data was tested with the Kolmogorov–Smirnov test with the use of histograms. We used the Levene’s test of homogeneity. Descriptive analysis was performed to compare baseline characteristics.

For continuous data: mean SD (parametric data) or medians and interquartile ranges (non-parametric data) were calculated. Differences were assessed using the Student’s *T*-test (parametric data) or the Mann–Whitney *U*-test (non-parametric data). Categorical data were compared using the Chi-square test. A *p-*value of < 0.05 was taken as the threshold of statistical significance.

## Results

A total of 150 procedures were included. The mean age of the patients was 43 years (± SD 16), 80 patients where male. The primary surgeon was an attending/consultant in 95 cases while a surgical resident supervised by a consultant performed the procedure in 55 cases.

In 81 of 150 procedures (54%), one or more adverse events occurred, ranging from 1 to 5 per procedure. In total, there were 147 adverse events, median 1 per procedure (IQR: 0.0–2.0). Most adverse events occurred in the joint replacement group (80%) (Table 1). In 53.9% of the procedures adverse events were associated with medical instruments or implants (53.9%). In 10.2% of the procedures instruments were not connected correctly and in 9.5% the instruments were not present in the OR at all. In 6.8% of the procedures the scrub nurse was not familiar with the procedure or the equipment.

**Table 1:** Different types of procedures, number of adverse events and median delay in min

Type procedure	Number of procedures with adverse events	%	Median delay per procedure (min)	IQR (min)
1. Osteosynthesis	33	67	13.0	4.2–45.5
2. Arthroscopy	16	47	10.0	1.7–16.0
3. Removal of hardware	11	50	10	5.0–15.0
4. Joint replacement	8	80	7.7	1.8–23.8
5. Bone grafting	6	75	7	2.0–40.0
6. Other	7	26	5.0	4.0–10.5
Total	81	54	10.0	4.2–20.0

Absence of OR staff was a factor contributing significantly to prolongation of procedures; for surgeons a median delay of 12.5 min, Table 2, and for anaesthesiologists a median delay of 6.0 min. In this study, the scrub nurses were never absent. 6 Procedures were delayed due to late arrival of a C-arm technician, median delay 10.0 min (IQR: 4.7–20.0).

**Table 2:** Delay per adverse event category in min

Adverse event category	Number of adverse events per category	%	Median delay per adverse event (min)	IQR (min)	Range (min)
1. Patient factors	6	4	40.0	25.5–48.7	12–60
2. Staff factors	24	16	5.0	0–15.0	0–45
3. Anaesthesia	13	9	7.0	1.5–17.5	0–60
4. Imaging equipment	20	14	6.5	3.0–10.0	0–20
5. Instruments/implants	79	54	0	0–0.4	0–45
6. OR equipment	5	3	5.0	0–7.5	0–10
Total	147		0.3	0–7.0	

In five of the 150 cases there could have been a negative side effect for the patient:

- Insufficient anaesthesia;
- Delayed administration of antibiotics (2 times);
- Twisted drill guide (too much of the femur was removed during an arthroplasty of the knee);
- Problems with sterility of instruments.

Thirty-six percent of the procedures were prolonged due to these adverse events. The total delay per procedure ranged between 30 s and 77 min.  Median delay was 10.0 min (IQR: 4.1–20.0). Bone grafting was the procedure contributing the most delay with 62.5% of the procedures being delayed due to adverse events. Osteosynthesis was the procedure contributing the highest median delay, 13.0 min (IQR: 4.2–45.5 min) (Table 1).

113 patients were operated under general anaesthesia. In 9.7% of those procedures the anaesthesia was insufficient. The surgical procedure had to be temporary terminated until the patient received sufficient anaesthesia to continue the procedure. Thirty-six patients had spinal (18/150) or loco-regional (18/150) anaesthesia. In 6 out of 36 patients this anaesthesia was insufficient. 146 out of 147 adverse events were not described in the operation report (99.3%). Only one report did mention the insufficiency of the loco-regional anaesthesia.

There was no significant difference in adverse events between elective and acute surgery, *p* = 0.71. Most adverse events occurred in the inpatient operating room (61.3%), compared to day surgery center surgery (46.6%), *p* < 0.05.

## Discussion

In 54% of the procedures 1–5 adverse events occurred. Most adverse events occurred in the category instruments and implants, followed by staff depending factors.

In most cases the equipment was malfunctioning or not present in the OR at all. This occurred due to failure to report earlier problems with the instruments, failure to resolve reported adverse events, failure to report missing instruments in sterilised material and inadequate check up and maintenance of equipment at the sterilisation unit.

In 9.5% of the procedures equipment was not present in the OR. This caused distraction of the surgeon, in some cases this led to prolongation of the procedure. Most adverse events in the category instruments and implants may theoretically be precluded by schooling of staff. Absence of staff could be caused by understaffing or by logistic reasons: sometimes the imaging equipment was requisite in two different OR’s at the same time.

Insufficient anaesthesia is an adverse event which could be caused by patient depending factors, e.g. habituation with medication, physiological factors, but by staff factors as well: for example inexperience of the anaesthesiology staff. The administration of antibiotics on time could be caused by the same factors  e.g. patient or staff depending factors. Adverse events regarding unsterilized instruments or dysfunctional instruments are unacceptable.

Regularly performed checks on the instruments, by example at the start of the procedure could potentially prevent these negative side effects.

In 99.3% of the procedures adverse events were not mentioned in the operation report. A higher rate of reporting of adverse events may enable the administration of adverse events resulting in a higher awareness of the incidence of adverse events.

De Vries et al. showed a decrease in complications following introduction of the SURPASS [6]. This checklist covers not only the surgical procedure, but the complete logistic procedure during the admittance as well. However, the SURPASS does not include a check of surgical instruments and implants, the main cause of adverse events in this study. Sterilised equipment is generally opened after the Time out-procedure. During this procedure a question about the availability of all the required equipment is generally asked, while a check of the equipment is not yet performed. This may explain the high incidence of adverse events in this study.

We clarified the need for specified checklists for instruments and implants prior to the start of trauma orthopedic surgical procedures. In our opinion specified checklists, which also have to include a check of surgical instruments and implants, will possibly reduce the incidence of adverse events.

## Conclusion

This cross-sectional observational study of 150 trauma orthopedic surgical procedures shows an incidence of adverse events of 54%. Most of the adverse events can easily be prevented. Future studies on the value of specific checklists for instruments and implants are necessary.
